# Micro-management of pluripotent stem cells

**DOI:** 10.1007/s13238-013-0014-z

**Published:** 2014-01-28

**Authors:** Wen-Ting Guo, Xi-Wen Wang, Yangming Wang

**Affiliations:** Peking-Tsinghua Center for Life Sciences, Institute of Molecular Medicine, Peking University, Beijing, 100871 China

**Keywords:** stem cells, miRNA, pluripotency, reprogramming

## Abstract

Embryonic and induced pluripotent stem cells (ESCs and iPSCs) hold great promise for regenerative medicine. The therapeutic application of these cells requires an understanding of the molecular networks that regulate pluripotency, differentiation, and de-differentiation. Along with signaling pathways, transcription factors, and epigenetic regulators, microRNAs (miRNAs) are emerging as important regulators in the establishment and maintenance of pluripotency. These tiny RNAs control proliferation, survival, the cell cycle, and the pluripotency program of ESCs. In addition, they serve as barriers or factors to overcome barriers during the reprogramming process. Systematic screening for novel miRNAs that regulate the establishment and maintenance of pluripotent stem cells and further mechanistic investigations will not only shed new light on the biology of ESCs and iPSCs, but also help develop safe and efficient technologies to manipulate cell fate for regenerative medicine.

## Introduction

Pluripotent stem cells (PSCs), including ESCs and iPSCs, can self-renew indefinitely while maintaining full developmental potential to produce any cell type (NIH, 2009; Buganim et al., 2013). These remarkable properties hold great potential for clinical applications in regenerative medicine. In particular, iPSCs generated from patient-specific somatic cells provide promise for personalized cell therapy, which could avoid immune rejection and the ethical issues associated with human ESCs. To fully realize their potential and avoid safety issues, proper strategies must be developed to control the self-renewal and differentiation of PSCs; this requires an understanding of the molecular details underlying these processes. Important regulatory modules in ESCs include signaling pathways, transcription factors, epigenetic factors, and noncoding RNAs (Melton and Blelloch, 2010; Ng and Surani, 2011; Wang and Blelloch, 2011; Watanabe et al., 2013). The dynamic interplay among these modules controls the switch between the pluripotent/self-renewal state and the differentiated state by regulating gene expression at the level of chromatin state, transcription, post-transcription, and post-translation.

MiRNAs are a class of small (18–25 nucleotides) regulatory non-coding RNAs (Bartel, 2009). With some exceptions (Vasudevan et al., 2007; Eiring et al., 2010), miRNAs regulate gene expression at the post-transcriptional level by inhibiting protein translation and/or reducing mRNA stability. Most mature miRNAs are produced through two sequential steps: cleavage of a long pri-miRNA ranging from hundreds to thousands of nucleotides to a hairpin pre-miRNA of ~ 70 nucleotides by DROSHA/DGCR8 in the nucleus and then cleavage to the mature miRNA by DICER in the cytoplasm (Kim et al., 2009b; Winter et al., 2009). The mature miRNA is then incorporated into the RNA-induced silencing complex (RISC) to regulate gene expression. Genomic studies have revealed that a single miRNA can regulate hundreds of targets (Lewis et al., 2005; Helwak et al., 2013). In addition, >60% of human genes are predicted to be miRNA targets (Friedman et al., 2009), suggesting the large potential of these RNAs in shaping the scope of gene expression. The large number of targets regulated by a single miRNA is due to their imperfect target recognition, often only requiring partial complementarity in the seed sequence (positions 2–8 at the 5′ end). Because of their ability to simultaneously control the expression of a large number of genes, miRNAs are well-positioned as master regulators to maintain or switch cell fate.

Deletion of proteins in the miRNA biogenesis pathway, such as DGCR8, DROSHA, or DICER, results in the loss of miRNAs in mammalian cells (Kanellopoulou et al., 2005; Murchison et al., 2005; Wang et al., 2007; Wu et al., 2012). These global miRNA-knockout cells provide invaluable reagents to tackle the functions of miRNAs, as the functional characterization of miRNAs is often complicated by the redundancy of miRNA families (Miska et al., 2007). ESCs with *Dgcr8* or *Dicer* knockout proliferate at a slower rate, with a slight accumulation of cells in G_1_ phase, and when induced to differentiate, cannot silence the self-renewal program, suggesting important roles of miRNAs in controlling the self-renewal and differentiation of ESCs (Kanellopoulou et al., 2005; Murchison et al., 2007). In addition, various studies have established miRNAs as important regulators during the generation of iPSCs. Functionally, these miRNAs can be categorized into five groups: those that regulate ESC cell cycle, stabilize the pluripotent state, silence the pluripotent state, promote reprogramming, and repress reprogramming. In this review, we summarize recent findings on miRNAs acting as essential regulators of ESCs and somatic cell reprogramming based on these categories.

## miRNAs that Regulate the ESC Cell Cycle

**Figure 1:**
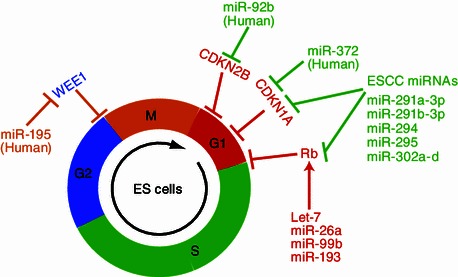
miRNAs that regulate cell cycle of mouse or human ESCs

ESCs have an unusual cell-cycle structure with an extremely short G_1_ phase (Wang and Blelloch, 2009). In the mouse, this is due to the constitutively-active Cdk2-Cyclin E complex which causes the hyperphosphorylation of the RB protein to maintain high activity of E2F1, a transcription factor promoting G_1_/S transition (Savatier et al., 1996; Stead et al., 2002; White and Dalton, 2005). Interestingly, *Dgcr8*-knockout mouse ESCs proliferate slowly with more cells accumulating in G_1_ phase (Wang et al., 2007), suggesting a function of miRNAs in shaping the ESC-specific cell-cycle structure. Screening using chemically-synthesized miRNA mimics has identified 11 miRNAs that promote the proliferation of *Dgcr8*-knockout ESCs (Wang et al., 2008). All but one (miR-19a) of the screening positive miRNAs have a similar seed sequence “AAGUGCU” or “AAAGUGC”. Four members of the miR-290-295 cluster and four members of the miR-302 cluster share the seed sequence “AAGUGCU” (Table 1). More interestingly, these miRNAs are highly expressed in ESCs and are quickly downregulated upon ESC differentiation (Houbaviy et al., 2003; Babiarz et al., 2008), suggesting a specific function of these miRNAs in ESCs. Consistent with their important roles in ESC biology, the transcription of these miRNAs is regulated by pluripotency factors such as OCT4, SOX2, and NANOG (Marson et al., 2008), and have recently been shown to be regulated by the super-enhancer that is formed by master transcription factors and the mediator (Whyte et al., 2013). Because of their roles in regulating the G_1_/S transition of the ESC cell cycle, they are named ESCC miRNAs for Embryonic Stem cell specific Cell Cycle regulating miRNAs (Fig. 1).

**Table 1:** miRNAs that regulate ESC cell cycle

miRNA	Seed sequence	Function	Targets	Species	Reference
miR-291a-3p, miR-291b-3p, miR-294, miR-295, miR-302a-d	AAGUGCU	Promoting G_1_/S transition at normal culture condition or cytostatic conditions	Cdkn1a, Rb1, Rbl2, Lats2	Mouse	Wang et al., 2008; Wang et al., 2013b
miR-302a-d	AAGUGCU	Promoting G_1_/S transition	CCND1 and CCND2	Human	Card et al., 2008
miR-372	AAGUGCU	Promoting G_1_/S transition	CDKN1A	Human	Qi et al., 2009
miR-195	AGCAGCA	Promoting G_2_/M transition	WEE1	Human	Qi et al., 2009
miR-92b	GGGACGG	Promoting G_1_/S transition	CDKN2B	Human	Sengupta et al., 2009

ESCC miRNAs target many inhibitors of the G_1_/S transition including Cdkn1a (also called p21), Lats2, Rb1, and Rbl2. Rbl1, another member of the Rb family, is also inhibited indirectly by ESCC miRNAs (Wang et al., 2008, 2013b). These results strongly suggest that ESCC miRNAs promote the G_1_/S transition and proliferation by inhibiting the Rb family. However, triple-knockout of Rb family proteins in the *Dgcr8*-knockout background neither increases the proliferation nor reduces the accumulation of cells in G_1_ phase (Wang et al., 2013b). These data raise questions about the exact function of repressing Rb family proteins by ESCC miRNAs in ESCs. Interestingly, further investigation indicates that repression of the Rb family proteins accounts for another important characteristic of ESCs, the lack of G_1_ restriction point (Schratt et al., 2001; Blagosklonny and Pardee, 2002; Wang et al., 2013b). Like cancer cells, ESCs do not accumulate in G_1_ phase under serum starvation or at cellular confluence. However, *Dgcr8*-knockout ESCs significantly accumulate in G_1_ phase (up to 60% under contact-inhibition conditions). ESCC miRNAs and triple *Rb* knockout effectively rescue this dramatic phenotype. Therefore the repression of Rb family proteins by ESCC miRNAs contributes to the lack of G1 restriction point in ESCs (Wang et al., 2013b).

MiRNAs are also important for proliferation and cell-cycle regulation in human ESCs. Qi et al. identified miR-372 that promotes the G_1_/S transition by targeting CDKN1A (Qi et al., 2009). The same study also identified miR-195 that promotes the G_2_/M transition by suppressing the G_2_/M checkpoint kinase WEE1 in human ESCs. This regulation is likely to be specific to human ESCs, as miR-195 is not highly expressed in mouse ESCs. In addition, Sengupta et al. identified miR-92b that promotes the G_1_/S transition by targeting the Cdk2-Cyclin D complex inhibitor Cdkn2b (also called p57) (Sengupta et al., 2009). Interestingly, not only the Cdk inhibitors but also Cdk4 and Cyclin D1 are repressed by miR-302 in human ESCs (Card et al., 2008). However, the function of this seemingly negative regulation of cell-cycle progression is not clear. Based on the recent discovery that Cdk2/Cyclin D activity is essential for the self-renewal of human ESCs and suppresses differentiation into the endoderm lineage by repressing TGF-beta-SMAD2/3 transcriptional activity (Pauklin and Vallier, 2013), this regulation likely monitors the differentiation process of human ESCs.

## miRNAs that Silence the Pluripotency Program

**Figure 2:**
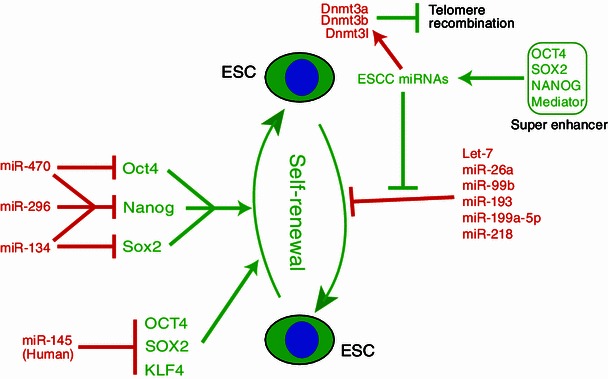
miRNAs that regulate the pluripotency and differentiation of mouse and human ESCs

*Dgcr8*-knockout ESCs do not silence their self-renewal under differentiation conditions, therefore ESCs require miRNAs to silence the pluripotency program. A direct way to silence the pluripotency program is to repress the pluripotency transcription factors such as Oct4, Sox2, and Nanog (Table 2, Fig. 2). Indeed, Tay et al. identified three MiRNAs (miR-134, miR-296, and miR-470) that inhibit the expression of these genes (Tay et al., 2008a, 2008b). miRNAs usually regulate gene expression through the 3′ untranslated regions (3′UTRs) of target mRNAs. However, the authors found that these miRNAs repress the expression of pluripotency factors by binding to their coding regions. This study for the first time demonstrated that regions other than 3′UTRs can be recognized and regulated by miRNAs. In fact, this type of regulation may be widespread for many miRNAs as revealed by recent large-scale studies (Helwak et al., 2013). Furthermore, this study demonstrated that species-specific interactions can also be functionally important, since four out of five identified targeting sites are not conserved in human and rhesus. Echoing this study, Xu et al. identified miR-145 that specifically binds the 3′UTR of human OCT4 to repress pluripotency in human ESCs (Xu et al., 2009). In addition, miR-145 also directly regulates two other pluripotency transcription factors, SOX2 and KLF4. The miR-145 binding sites in the 3′UTRs of SOX2 and KLF4 are conserved in mice, suggesting that this miRNA also regulate pluripotency in mouse ESCs.

**Table 2:** miRNAs that silence ESC self-renewal

miRNAs	Seed sequence	Upregulated in differentiation	Targets	Species	Reference
Let-7	GAGGUAG	-LIF, -LIF + RA	nMyc, Sall4, Lin28a	Mouse	Melton et al., 2010
miR-134	GUGACUG	-LIF, -LIF + RA	Nanog, Sox2, Lrh1	Mouse	Tay et al., 2008a; Tay et al., 2008b
miR-296	GGGCCCC	-LIF, -LIF + RA	Nanog	Mouse	Tay et al., 2008a
miR-470	UCUUGGA	-LIF, -LIF + RA	Oct4, Nanog	Mouse	Tay et al., 2008a
miR-145	UCCAGUU	-LIF, -LIF + RA	OCT4, SOX2, KLF4	Human	Xu et al., 2009
miR-26a	UCAAGUA	-LIF, -LIF + RA	Unknown	Mouse	Wang et al., 2013b
miR-99b	ACCCGUA	-LIF, -LIF + RA	Unknown	Mouse	Wang et al., 2013b
miR-193	GGGUCUU	-LIF, -LIF + RA	Unknown	Mouse	Wang et al., 2013b
miR-199a-5p	CCAGUGU	-LIF, -LIF + RA	Unknown	Mouse	Wang et al., 2013b
miR-218	UGUGCUU	-LIF, -LIF + RA	Unknown	Mouse	Wang et al., 2013b

Another pluripotency-silencing miRNA is let-7, one of the founding and the most conserved members of the miRNA family (Pasquinelli et al., 2000; Reinhart et al., 2000). It was first discovered as a heterochronic gene to control the switching of developmental timing in *Caenorhabditis elegans*. Loss of let-7 by mutation leads to the reiteration of larval cell fates during the adult stage, and an increased let-7 gene dosage leads to the precocious expression of adult fates during larval stages. The human and mouse let-7 family contains 9 closely-related miRNAs (with the seed sequence “GAGGUAG”) distributed over 8 and 7 chromosome locations, respectively. Interestingly, let-7 is repressed in ESCs but rapidly upregulated during ESC differentiation (Newman et al., 2008; Viswanathan et al., 2008). The expression of let-7 remains high in a variety of somatic cells. Consistent with its role in enabling differentiation, the introduction of let-7 into *Dgcr8*-knockout ESCs successfully silences their self-renewal and pluripotency program (Melton et al., 2010). More interestingly, although mature let-7 is not expressed, primary transcripts of let-7 are expressed even in undifferentiated ESCs (Newman et al., 2008; Viswanathan et al., 2008). The production of mature let-7 is blocked by the RNA-binding protein LIN28 at the step of DICER processing. LIN28 recruits the terminal uridine transferase TUT4 to add a string of uridines to the 3′ end of pre-let-7, which then triggers its degradation by the exonuclease Dis3l2 (Heo et al., 2009, 2012; Thornton et al., 2012; Chang et al., 2013). The evolution of such a complicated post-transcriptional regulatory program instead of shutting down the transcription is not clear. However, by keeping the precursor let-7 readily available, ESCs could respond quickly to differentiation cues to produce the mature let-7.

During ESC differentiation, numerous miRNAs are upregulated (Wang et al., 2013b; Marson et al., 2008; Bar et al., 2008). However, whether all these miRNAs play a causal role in inducing differentiation is not known. To systematically identify miRNAs that silence the pluripotent state, an unbiased screen was designed based on the impact of transfected miRNAs on *Dgcr8*-knockout ESCs (Wang et al., 2013b). From a library of 250 miRNA mimics, 32 miRNAs were found to silence the pluripotency program (AP activity decreased 75% or more). Fourteen of these miRNAs were confirmed to be upregulated in two differentiation conditions: in the absence of leukemia inhibitory factor or in the presence of retinoic acid. Consistent with previous reports, these miRNAs include miR-134 and miR-145 (Tay et al., 2008a, 2008b; Xu et al., 2009). More interestingly, five other miRNAs (miR-26a, miR-99b, miR-193, miR-199a-5p, and miR-218) are able to silence ESC self-renewal. These miRNAs have different seed sequences and are expressed in different cell lineages (Landgraf et al., 2007). It remains a mystery whether these miRNAs function through a common mechanism or induce different differentiation programs in various contexts. Evaluating the targets and pathways regulated by these miRNAs is required to understand how they exert their self-renewal silencing function.

During ESC differentiation, the pluripotency program is shut off and a differentiated program is established. This is accompanied with the switch of cell cycle structure (Wang et al., 2013b; Savatier et al., 1996). At the onset of differentiation, the G_1_ phase of cell cycle is gradually elongated and cells start to accumulate in G_0_/G_1_ phase. MiRNAs silencing ESC self-renewal are clearly involved in the switch of cell cycle structure, as Let-7, miR-26a, miR-99b, miR-193, and miR-218 significantly arrested cells in the G_1_ phase (Wang et al., 2013b). In addition, the ESCC miRNAs oppose the G_1_ arrest induced by these miRNAs, indicating ESCC miRNAs maintain the unique ESC cell cycle structure at multiple conditions. This opposing regulation of the G_1_ arrest is at least in part dependent on the RB proteins, since knocking out three RB family proteins partially rescued the G_1_ arrest induced by four differentiation-inducing miRNAs (Let-7, miR-26a, miR-99b, and miR-193) in the *Dgcr8* knockout ESCs. How differentiation-inducing miRNAs activate the RB pathway is unknown. Systematic dissection of functional targets is required to reveal the answer in future.

## miRNAs that Stabilize the Pluripotency Program

Interestingly, the let-7, miR-26a, miR-99b, miR-193, miR-195a-5p, and miR-218 are found to silence the ESC self-renewal only in the *Dgcr8* knockout but not wild type background (Wang et al., 2013b; Melton et al., 2010), suggesting some miRNAs in wild type ESCs block the function of these miRNAs. Indeed, co-introduction of highly ESC enriched ESCC miRNAs successfully prevented the silencing of self-renewal by the differentiation-inducing miRNAs (Fig. 2). How these miRNAs stabilize the pluripotency program in the presence of differentiation-inducing miRNAs is not clear. In the case of let-7–miR-294/302 antagonism, these miRNAs opposingly regulate the level of Sall4 and c-Myc. More interestingly, transfection of miR-294/302 upregulates the expression of Lin28a (Melton et al., 2010), which then negatively regulates the maturation of let-7 family of miRNAs (Newman et al., 2008; Viswanathan et al., 2008). Therefore miR-294/302 family blocks the function of let-7 at multiple levels. However, because other differentiation-inducing miRNAs have different seed sequences and seem not to regulate the Sall4 and c-Myc directly; miR-294/302 may act through some unidentified common pathways to antagonize these miRNAs. An interesting hypothesis suggests the possible role of cell-cycle-regulating pathways, as the ESCC miRNAs and several differentiation-inducing miRNAs opposingly regulate the G_1_/S transition through the RB pathway. However, triple knockout of *Rb* family did not have any effects in blocking the silencing of self-renewal by differentiation-inducing miRNAs (Wang et al., 2013b). Therefore miR-294/302 family of miRNAs likely regulates the cell cycle and self-renewal through separate mechanisms. Future studies are required to identify other pathways responsible for the self-renewal-promoting function of miR-294/302.

Another interesting function of the miR-294/302 family of miRNAs is to control global DNA methylation through indirectly activating the transcription of DNA methyltransferases Dnmt3a, Dnmt3b, and Dnmt3l (Benetti et al., 2008; Sinkkonen et al., 2008), although this regulation may play little role in its pluripotency-stabilizing function. A possible direct target underlying this function is the transcriptional repressor Rbl2. DNA methylation is important for a variety of cellular processes such as transposon silencing, telomere recombination, and the silencing of gene expression (Curradi et al., 2002; Irvine et al., 2002; Gonzalo et al., 2006; Huang et al., 2012). It is found that the miR-294/302 keeps the high level of methylation at the subtelomeric region to limit telomere recombination, therefore controlling the abnormal elongation of telomeres in ESCs (Bentti et al., 2008). More interestingly, the miR-294/302 was shown to be important for the methylation of the Oct4 promoter in ESCs under a differentiation condition (-LIF + RA) (Sinkkonen et al., 2008). This observation was counterintuitive as this function is against the role of miR-294/302 in stabilizing the pluripotency. However, it may not be surprising since the opposite function for some protein coding genes is observed under different biologic contexts (Lin et al., 1999; Boxer and Dang, 2001; Massagué, 2012). It is worth to note that these studies were carried out in *Dicer*-knockout ESCs and are controversial based on a recent study (Ip et al., 2012). As DICER processes various types of small RNAs other than miRNAs and has been shown to be important for the genomic stability (Murchison et al., 2007; Kanellopoulou et al., 2005), independent investigations using miRNA-specific-knockout ESCs (e.g. Dgcr8 knockout) may help resolve the discrepancy.

The miR-200 family is another pluripotency-stabilizing miRNA family which contains 5 members (miR-200c/141 and miR-200b/200a/429) located at two genomic loci (Kozomara and Griffiths-Jones, 2011). These miRNAs are expressed in ESCs but downregulated during ESC differentiation (Lin et al., 2009; Gill et al., 2011). The miR-200 family is transcriptionally regulated by c-Myc, and the regulation seems to be specific for ESCs as the overexpression of c-Myc does not change the expression of miR-200 family in ESC-derived-hematopoietic stem cells or mixed B/T cell lymphomas. Interestingly, the overexpression of miR-200 family attenuates the downregulation of pluripotency factors in ESCs under the culture condition without LIF, suggesting these miRNAs block the differentiation of ESCs. Close inspection indicates that the overexpression of miR-200 family traps the ESCs at the epiblast stem cell (EpiSC) stage (Gill et al., 2011), a primed pluripotent state that are thought to be more similar to human ESCs. The essential targets of miR-200 include Zeb1 and Zeb2, two key transcription factors promoting the EMT process (Burk et al., 2008; Gregory et al., 2008; Korpal et al., 2008; Park et al., 2008). Since EMT plays important roles during ESC differentiation (Eastham et al., 2007; Spencer et al., 2007; Martínez-Estrada et al., 2010), miR-200 may modulate ESC differentiation through regulating the EMT process.

## miRNAs that Promote or Suppress Reprogramming

iPSCs are pluripotent stem cells generated by de-differentiating somatic cells using defined factors (Takahashi and Yamanaka, 2006; Hanna et al., 2010; Okita and Yamanaka, 2011). Initially the reprogramming is only enabled by virally introducing pluripotency factors Oct4, Sox2, Klf4, and c-Myc. However, the technology has been improved significantly since the invention. For example, the integration-free induction strategies using *in vitro*-synthesized mRNAs, proteins or episomal vectors avoid the potential danger from the integration of viral sequence into the host genome (Kim et al., 2009a; Zhou et al., 2009; Warren et al., 2010; Okita et al., 2011). More excitingly, Hou et al. recently reprogrammed mouse fibroblasts to iPSCs with only small molecules (Hou et al., 2013). The key objectives in studying reprogramming include enhancing reprogramming efficiency, understanding the molecular mechanisms of reprogramming, and more importantly improving the quality of iPSCs, which is important for their potential therapeutic applications. Various studies have shown that miRNAs can be manipulated to improve the efficiency of reprogramming and potentially the quality of iPSCs. Because miRNAs can be introduced into cells through the transfection of chemically-synthesized double-strand RNAs, the reprogramming approach based on miRNAs are naturally safer than using virus or DNA vectors that could interfere with the genome of targeted cells. In addition, as we discuss below, dissecting miRNA targets could also provide insights on the mechanism of reprogramming process.

**Figure 3:**
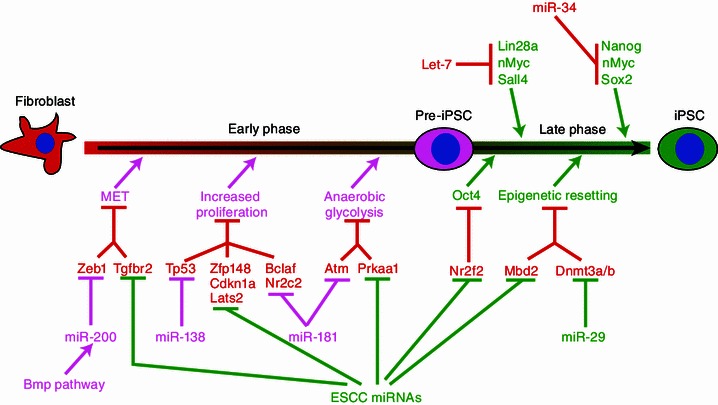
miRNAs that promote or suppress somatic reprogramming

MiR-294/302 family of miRNAs is the first example that miRNAs can promote the mouse iPSC generation (Judson et al., 2009). This may not be surprising since these miRNAs stabilize the pluripotency state of ESCs. Interestingly, these miRNAs promoted the reprogramming of mouse fibroblasts along with Oct4, Sox2, and Klf4 (OSK) at both the early and later stages (Table 3 and Fig. 3). It is amazing that a single miRNA promotes both stages as different pathways are required to be shut down during the early and later stages of reprogramming. Later the miR-294/302 has also been shown to promote the generation of human iPSCs (Subramanyam et al., 2011). Mechanistically, the miR-294/302 seems to act at multiple levels to break barriers towards the pluripotency. These barriers include the epithelial to mesenchymal transition, cell cycle, cell death, and mitochondrial function (Judson et al., 2013).

**Table 3:** miRNAs that promote or suppress somatic reprogramming

miRNAs	Seed sequence	Targets	Function in reprogramming	Species	Reference
miR-291a-3p, miR-291b-3p, miR-294, miR-295, miR-302a-d, miR-372	AAGUGCU	Tgfbr2, RhoC, Mbd2, Nr2f2, Lefty, Cdkn1a, Brp44 l, Zfp128, Hivep2, Hipk3, Ddhd1, Dpysl2, Pten, Cfl2, 9530068E07Rik	Promotion	Mouse Human	Judson et al., 2009; Subramanyam et al., 2011; Liao et al., 2011; Judson et al., 2013; Lee et al., 2013
miR-17-92	AAAGUGC	Tgfbr2, p21	Promotion	Mouse	Li et al., 2011
miR-138	GCUGGUG	TP53	Promotion	Mouse	Ye et al., 2012
miR-181	ACAUUCA	Bptf, Lin7c, Tox, Cpsf6, Dnaj13, Nr2c2, Bclaf1, Ywhag, Nol8, Igh2bp2, Marcks, Cdyl	Promotion	Mouse	Judson et al., 2013
miR-29b	AGCACCA	Dnmt3a, Dnmt3b	Promotion	Mouse	Guo et al., 2013
miR-34a	GGCAGUG	Nanog, Sox2, nMyc	Repression	Mouse	Choi et al., 2011
miR-21	AGCUUAU	P85alpha, Spry1	Repression	Mouse	Yang et al., 2011
miR-29a	AGCACCA	Cdc42, p85alpha, Spry1	Repression	Mouse	Yang et al., 2011
miR-766	CUCCAGC	SIRT6	Repression	Human	Sharma et al., 2013

### MiRNAs that regulate p53 pathway

Consistent with miRNAs functioning to regulate a variety of biologic pathways, many different miRNAs have been shown to promote the iPSC generation through overcoming different barriers toward reprogramming. Activation of the p53 pathway triggers cell cycle arrest, senescence or cell death. During reprogramming, Klf4 and c-Myc upregulate the expression of p53, a potential beneficial response by cells to maintain the genomic integrity under stress. Knocking down p53 significantly promoted the iPSC generation, likely due to the increase of cell numbers (Zhao et al., 2008; Hong et al., 2009; Kawamura et al., 2009; Li et al., 2009; Marión et al., 2009; Utikal et al., 2009). However, the complete block of the p53 pathway could cause genomic instability therefore affecting the quality of iPSCs. On the other hand, fine-tuning p53 expression may promote the reprogramming efficiency without dampening the quality of iPSCs. Ye et al. predicted and confirmed that miR-138 can promote the iPSC generation through regulating p53 expression (Ye et al., 2012). Interestingly, the upregulation of miR-138 seems to be required for iPSC generation in that context. Moreover, miR-138 may not reduce the quality of iPSCs based on functional tests such as teratoma formation and the activation of an imprinting locus *Dlk-Dio3* region. In addition, targeting other inhibitors along the p53 pathway is also beneficial for the reprogramming process. For example, miR-294/302 and the miR-17-106-92 cluster have been shown to repress Cdkn1a and Rb family genes to promote the iPSC formation (Judson et al., 2009; Li et al., 2011).

### MiRNAs that promote MET

Genomic and genetic studies as well as the pure observation of morphologic changes during the iPSC induction process suggest that mesenchymal-to-epithelial transition (MET) or blocking the opposite process epithelial-to-mesenchymal transition (EMT) is required for the reprogramming (Li et al., 2010; Samavarchi-Tehrani et al., 2010). The epithelial or mesenchymal cell fate is controlled by a variety of signaling pathways such as BMP and TGF-beta signaling pathways as well as key transcription factors including Zeb1/2, Snai1/2 and Twist1/2 (Acloque et al., 2009; Kalluri, 2009; Kalluri and Weinberg, 2009; Zeisberg and Neilson, 2009; Massagué, 2012). Modulating these regulators is expected to increase the efficiency of reprogramming. Indeed the miR-200 family of miRNAs, containing miR-200a/b/c, miR-203, miR-205, and miR-141, represses the mesenchymal promoter Zeb1 and significantly promotes the reprogramming process (Samavarchi-Tehrani et al., 2010; Wang et al., 2013a). Likewise, the miR-294/302 family (the miR-372/302 family in human) and the miR-17-92 cluster increase the reprogramming efficiency by negatively regulating TGF-beta pathway (Li et al., 2011; Liao et al., 2011; Subramanyam et al., 2011).

### MiRNAs that regulate epigenetic factors

During the reprogramming, differentiated cells gradually lose the epigenetic signature of the somatic cell and adopt the epigenetic signature of the pluripotent stem cell (Maherali et al., 2007; Fussner et al., 2011; Hochedlinger and Plath, 2009; Buganim et al., 2013). This epigenetic reprogramming includes the resetting of histone modifications, DNA methylation, and chromatin conformations, which are catalyzed and regulated by a variety of enzymes and protein complexes. Many regulators such as H3K36 demethylases KDM2A/B and H3K9 methyltransferase SETDB1 are required for the reprogramming process (Wang et al., 2011; Liang et al., 2012; Onder et al., 2012), while several other regulators such as H3K9 methyltransferase SUV39h1 and H3K79 methyltransferase DOT1L inhibit the reprogramming process (Onder et al., 2012). miRNAs targeting these enzymes or regulators have been shown to modulate the reprogramming process. The ESCC MiRNAs have been found to repress the expression of methyl CpG binding protein MBD2; and knocking down MBD2 can increase the efficiency of reprogramming (Subramanyam et al., 2011; Lee et al., 2013). In addition, miR-29b is upregulated by SOX2 during reprogramming process and overexpressing miR-29b promotes the iPSC formation. This promotion is at least in part explained by the inhibition of Dnmt3a/b at the 3′UTR by miR-29b (Guo et al., 2013). Given that epigenetic regulators are important for the reprogramming and a large number of examples of miRNA-epigenetic factor regulation, more epigenetic factor-targeting miRNAs are expected to function as either the promoter or the barrier of the reprogramming process.

### Other miRNAs that promote reprogramming

To systematically identify miRNAs that can modulate the iPSC production, Judson et al. screened 570 chemically-synthesized mature mouse miRNA mimics for their ability to promote OSK-induced reprogramming of mouse embryonic fibroblasts (MEFs) (Judson et al., 2013). This study identified many previously reported iPSC-promoting miRNAs such as ESCC miRNAs, as well as several novel miRNAs including miR-181, miR-19a*, miR-30, miR-34*, miR-144, miR-324, miR-451, and miR-677. By testing a small set of miR-181 targets in the reprogramming, Judson et al. then showed that miR-181, much like miR-294/302, represses multiple targets involved in the EMT, cell death, cell cycle, and mitochondria function. Repression of these targets produces cooperative effects on the efficiency of reprogramming and the morphology of reprogrammed iPSC colonies. These data are consistent with miRNAs functioning through fine-tuning multiple targets to remove the molecular barriers that divert the reprogramming cells away from the pluripotency.

### MiRNAs that suppress reprogramming

MiRNAs are not only promoters but also barriers of the reprogramming process. The first example of such barriers is let-7 (Melton et al., 2010). The inhibition of let-7 successfully increased the reprogramming efficiency. Since the reprogramming requires the activation of some of let-7 targets such as Lin28a, and let-7 is broadly expressed in various differentiated cells, the let-7 might act as the reprogramming barrier by repressing the expression of genes that are important for PSCs. In addition, several studies have shown that let-7 regulates cell cycle and glucose metabolism (Legesse-Miller et al., 2009; Zhu et al., 2011; Wang et al., 2013b). Therefore it is likely that let-7 stabilize the differentiated cell fate to block reprogramming through multiple pathways. Another prominent example is the miR-34 family which contains miR-34a/b/c (Kozomara and Griffiths-Jones, 2011). Inhibition of members of miR-34 family, particularly miR-34a, significantly increases the efficiency of iPSC production (Choi et al., 2011). Again this miRNA function as the barrier to block reprogramming by targeting pluripotency factors and possibly also cell cycle regulators. In retrospect, it may not be coincidental and surprising that let-7 and miR-34 are barriers of the reprogramming process, since both miRNAs induce differentiation in ESCs (Melton et al., 2010; Choi et al., 2011; Jain et al., 2012; Wang et al., 2013b). However, given that miR-181 family acts as both differentiation-inducing and yet also reprogramming-promoting factors (O’Loghlen et al., 2012; Wang et al., 2013b), it would be interesting to test whether other differentiation-inducing miRNAs can act as the reprogramming inducer or barrier. Equally important is to perform unbiased inhibitor screen for the miRNAs that block the reprogramming. Such efforts will certainly deepen our understanding on the mechanism of the reprogramming process.

## Conclusion and Future Directions

In less than a decade, researchers have established miRNAs as important regulators during the maintenance and differentiation of ESCs as well as the establishment of iPSCs. These RNAs control proliferation, survival, cell cycle, and the pluripotency program of ESCs. They also serve as the barriers or the inducers during the reprogramming process. Systematic gain or loss of function screens are discovering more miRNAs playing important roles in ESCs and during reprogramming (Judson et al., 2013; Wang et al., 2013b). Advanced genomic approaches are paving the way for discovering functional miRNA targets and understanding how miRNAs execute their function (Hanina et al., 2010; Leung et al., 2011; Helwak et al., 2013). However, many important questions are still waiting to be answered. Since pluripotent stem cells exist in two different states (naïve and prime), do miRNAs play any roles in defining these two developmentally related states? What is the relationship between different miRNAs that stabilize or silence the pluripotency program? The opposing function between ESCC miRNAs and let-7 and maybe other differentiation-inducing miRNAs suggest that these miRNAs could form functional feedback networks. In addition, how is the transcription and maturation of these miRNAs regulated and incorporated into the pluripotency network? Stories about the super enhancer controlling the expression of miR-290 cluster and intensive post-transcriptional regulation on let-7 maturation (Newman et al., 2008; Viswanathan et al., 2008; Heo et al., 2009, 2012; Thornton et al., 2012; Chang et al., 2013; Whyte et al., 2013) suggest that novel mechanisms regulating miRNA expression could be discovered through studying various miRNAs that control the establishment and maintenance of the pluripotency program. Finally, what is the relationship between different targets or pathways regulated by a miRNA that controls the pluripotency program? Small scale analysis reveals that repression of different targets of miR-294/302 leads to cooperative effects on reprogramming (Judson et al., 2013). It is expected that more complex relationships between different targets will be discovered when large scale functional analysis of miRNA targets are performed. Answering these questions will not only offer huge insights for the mechanistic understanding of the establishment and maintenance of PSCs, but also help the development of safe and efficient technologies to manipulate the cell fate for regenerative medicine.

## Abbreviations

EMT: epithelial-to-mesenchymal transition
ESCs: embryonic stem cells
iPSCs: induced pluripotent stem cells
MEFs: mouse embryonic fibroblasts
MET: mesenchymal-to-epithelial transition
PSCs: pluripotent stem cells
RISC: RNA-induced silencing complex
